# The relativity of regulatory T cell suppression modes

**DOI:** 10.3389/fimmu.2026.1771099

**Published:** 2026-02-10

**Authors:** Lu Bai, Vivek Choudhary, Yongqiang Feng

**Affiliations:** Department of Immunology, St. Jude Children’s Research Hospital, Memphis, TN, United States

**Keywords:** antigen load, antigen-specific suppression, bystander suppression, immune tolerance, regulatory T cells, TCR repertoire

## Abstract

Foxp3-expressing CD4 regulatory T (Treg) cells are essential for maintaining immune tolerance through antigen-specific and bystander suppression modes. They carry a diverse T-cell antigen receptor (TCR) repertoire against a broad spectrum of antigens. The TCR repertoire acquired during Treg induction exhibits significant variations across antigens, leading to uncertainty in target coverage and representation. Accumulating reports indicate a remarkable complexity in the functional modes of Treg cells under various immunological contexts, presenting significant challenges for further investigation. In this article, we propose that the relative significance and capabilities of Treg suppression modes shift according to the levels of specific antigens and the resulting TCR signal strength. Testing the applicability of this model and identifying its modifiers would offer valuable insights for basic research and translational applications.

## Introduction

Treg cells are induced in the thymus or periphery, playing a dominant role in suppressing conventional T (Tcon) cells that mount inflammatory responses to self-antigens and foreign antigens in the periphery (1, 2). Forkhead box protein P3 (Foxp3) is exclusively expressed by Treg cells, acting as the master regulator of Treg fate and immune suppressive function (3–5). Over two decades of intensive research have yielded remarkable progress in understanding the immunological roles and underlying mechanisms of Treg cells across diverse contexts, thanks to the utilization of various animal models (6–8). However, the mechanisms by which Foxp3 regulates gene expression remain to be fully explored (9–11). Accumulating reports have unveiled the increasing complexity of Treg function in various immunological settings. This poses significant challenges in comprehending the fundamental principles governing intricate Treg function required for basic and translational research.

Several mechanisms are known to mediate the immunosuppressive function of Treg cells, directly or through antigen-presenting cells (APCs). However, since Treg cells possess a diverse TCR repertoire targeting numerous antigens and most research was conducted with polyclonal Treg cells with undetermined antigen specificity, the behaviors of Treg cells have been elusive, regarding the target specificity in suppressing Tcon cells. This introduces additional complexity to the mechanisms that mediate Treg function. The natural variations in Treg induction across antigens (12–19), which affects target coverage and representation, raise questions about the underlying principles and mechanisms by which T-cell immune tolerance is reliably maintained.

In a recent study, Klawon et al. explored prostate-specific Treg cells when the cognate antigen is presented at the endogenous level or delivered presumably at a higher level through engineered *Listeria monocytogenes*, revealing the capabilities of distinct Treg suppression mechanisms (20). These findings reignite the unresolved questions about the immunological contexts, determinants, capabilities, and limitations of Treg bystander and antigen-specific suppressions. In this article, we examine published reports and delve into a theoretical analysis. This leads to a coherent model in which the relative significance and capabilities of Treg suppression modes shift in response to the TCR signal strength (above the tonic or basal level) resulting from antigen load.

## Treg cells suppress Tcon cells through two distinct modes

Treg cells are induced in tolerogenic environments, such as thymic medulla and intestines upon antigen or agonist stimulation through unique APCs (21–27). This enables the adaptive immune system to develop a tolerance memory during initial encounter with self-antigens and harmless foreign antigens, such as those in food and commensal microbiome. The mechanisms by which Treg cells are exclusively induced by these specialized APCs remain to be fully elucidated. Treg cells are required to suppress Tcon cells throughout an animal’s lifespan (6). This process is believed to be overall antigen-specific, ensuring that the response of Tcon cells to unrelated antigens, such as pathogens, is not interfered with.

**Figure 1:**
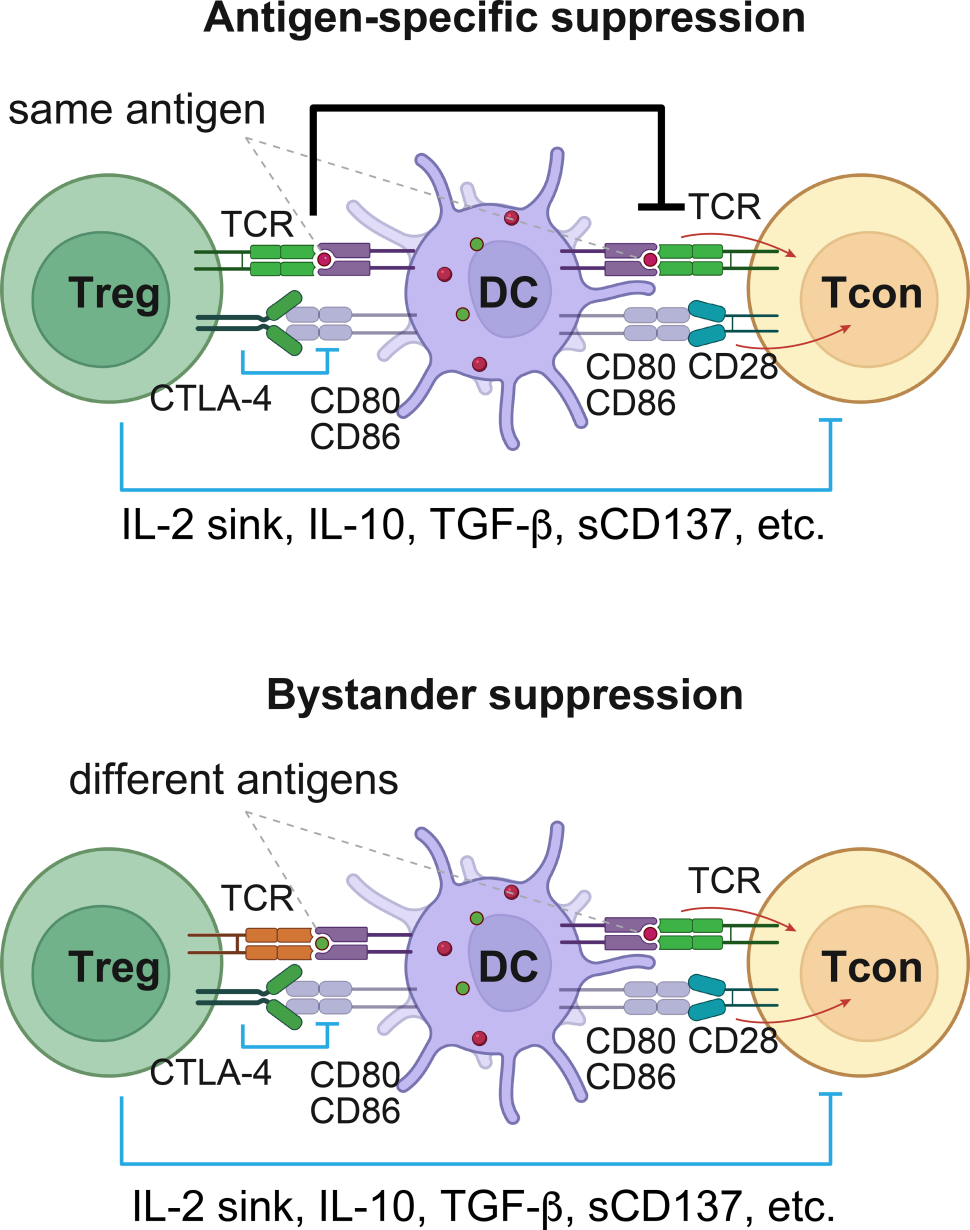
The two modes of suppression mechanisms employed by Treg cells. Tcon cells are restricted by Treg cells through various mechanisms, which can be categorized into antigen-specific and bystander suppression modes. Treg cells recognizing the same antigen as the Tcon cells exerts antigen-specific suppression (top). This is primarily accomplished through competition for antigen binding via overlapping TCR repertoires and the depletion of co-stimulatory receptors CD80/CD86 and peptide-MHC II on APCs. These antigen-engaged Treg cells also restrain the activation and proliferation of Tcon cells by depleting local IL-2 (IL-2 sink) through IL-2 receptor containing high-affinity subunit CD25, or secreting immune-suppressive molecules, such as IL-10, TGF-β, and soluble CD137 (sCD137). For simplicity, other factors that mediate the suppressive functions of Treg cells are not shown. These collectively suppress Tcon cells, where antigen-specific mechanisms are dominant. Polyclonal Treg cells, upon activation by different antigens, can also suppress Tcon cells of unrelated antigen specificity within proximity through these mechanisms, except for antigen competition, which is commonly referred to as bystander suppression (bottom). Notably, bystander suppression mode also relies on TCR signaling Treg cells received in the past. DC, dendritic cell. This figure was created using Biorender.com.

Treg cells suppress Tcon cells through various mechanisms, which can be categorized into two basic modes: antigen-specific and bystander suppressions (**Figure 1**). The antigen specificity of Treg-mediated immunosuppression is evident in three key features. First, Treg cells continuously rely on TCR signaling, likely exceeding tonic strength, to maintain their activated state and execute immune regulatory function (1, 28–30). Second, Treg cells possess an advantage over Tcon cells during antigen recognition. For example, they carry a TCR repertoire with a higher affinity for self-antigens (31). Treg cells also remain in an active state even in the absence of immune challenges. This is achieved through specialized regulatory circuits, which are partially dependent on Foxp3 but independent of TCR affinity (32–37). Third, during interaction with APCs, Treg cells deplete costimulatory receptors CD80/CD86 and peptide-major histocompatibility complex class II (MHC II) complexes on APCs (36, 38, 39). These, along with other mechanisms that require further investigation, allow Treg cells to surpass Tcon cells in antigen binding, thereby achieving antigen-specific suppression.

Treg cells also suppress Tcon cell activation and proliferation via extrinsic factors. For example, elevated expression of the high-affinity subunit of the interleukin (IL)-2 receptor, CD25, enables Treg cells to deplete local IL-2, serving as an IL-2 sink (40); Treg cells secrete soluble factors like transforming growth factor beta (TGF-β), IL-10, and soluble CD137 (sCD137, also known as 4-1BB or Tnfrsf9) (41), which are elevated after antigen stimulation, to suppress Tcon cells (8, 10); Treg cells express high-level ectonucleoside triphosphate diphosphohydrolase 1 (CD39) and ecto-5’-nucleotidase (CD73) that convert ATP to adenosine to activate adenosine receptors to suppress Tcon cells and other immune cells (42). These mechanisms, while also contributing to antigen-specific suppression, can potentially restrict Tcon cells that react to unrelated antigens presented by APCs in the same environment, known as Treg bystander suppression (**Figure 1**). Similarly, the downregulation of CD80/CD86 on APCs by Treg cells may potentially facilitate immune suppression across a wider range of antigenic stimuli.

Treg bystander and antigen-specific suppressions are interconnected. Antigen stimulation is required to maintain Treg survival, activation, and suppressive function. Though separated in space and time, it may promote Treg’s bystander suppression. The existence of these two distinct categories of suppression mechanisms raises questions about their interrelation, capabilities, limitations, and suitable contexts during Treg-mediated immune tolerance.

## Treg induction introduces variations in target coverage and representation

To gain insights into the interplay between Treg antigen-specific and bystander suppressions, it is important to examine the target coverage and representation of Treg cells. Treg cells carry a diverse TCR repertoire that targets an astronomical number of peptide antigens (43). Considering potential self-peptides (greater than 10^11^) and the number of Treg cells per mouse (less than 10^7^), the number of Treg cells would vary significantly for individual peptides. This variation would be more pronounced for peptides expressed at low levels by APCs during Treg induction or for peptides that trigger low-level TCR signaling, which is unfavorable for Treg induction.

**Figure 2:**
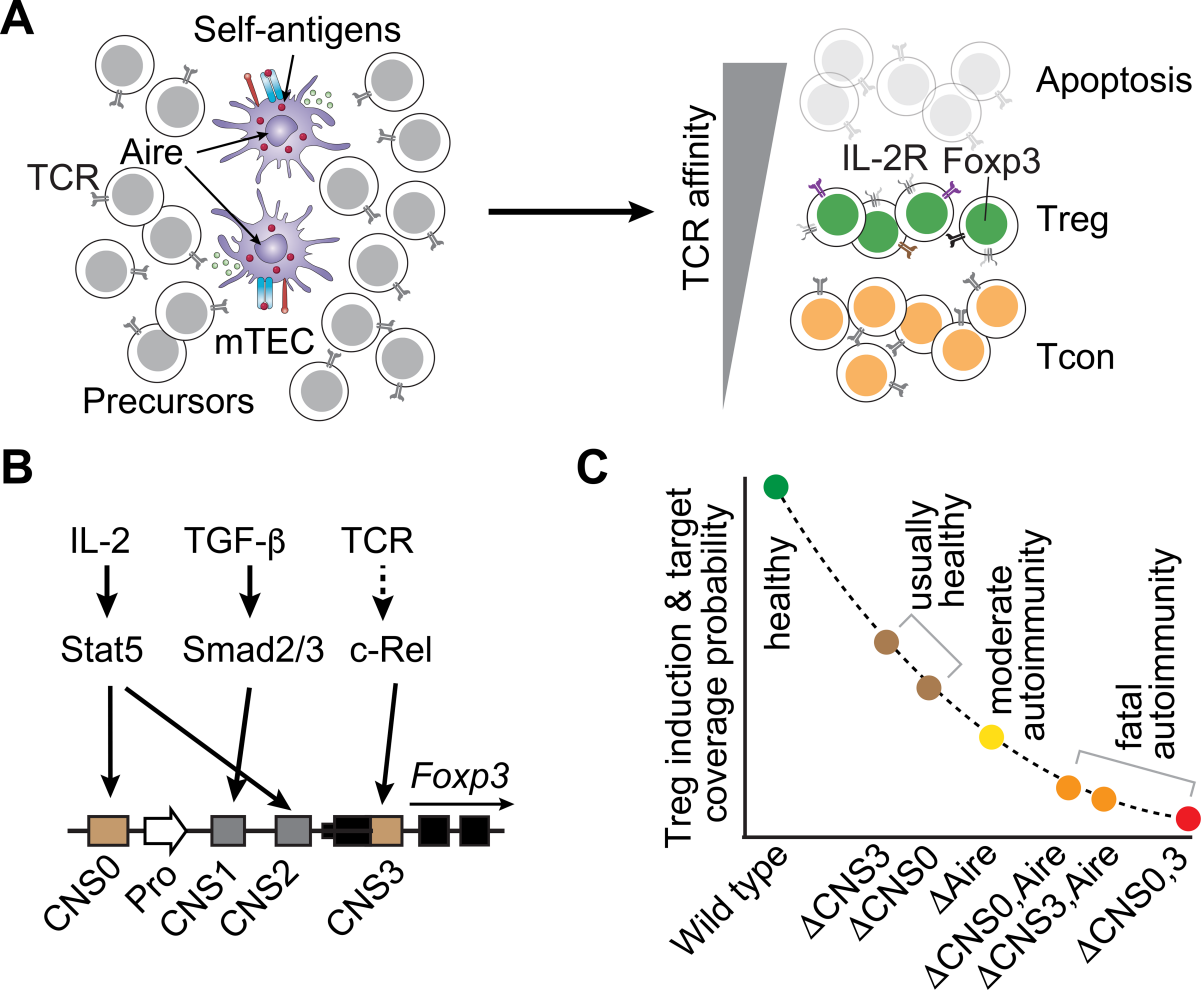
The relationship between the induction of polyclonal Treg cells and the capacity for immune tolerance. **(A)** A schematic diagram illustrates thymic T-cell selection and Treg induction. Aire is specifically expressed by mTECs to facilitate the expression of tissue-restricted self-antigens through a process known as thymic mimicry (21–23, 91). Precursor cells subjected to strong stimulation by self-antigens undergo apoptosis (thymic negative selection). Those receiving moderate stimulation differentiate into Treg cells. The remaining cells contribute to the Tcon cell pool. **(B)** Foxp3 induction during Treg development is facilitated by three enhancers CNS0, CNS1, and CNS3. These enhancers are regulated by Treg-inducing signals, as indicated by arrows. For simplicity, other factors interacting with these enhancers, such as transcription factors and epigenetic modifications, are not depicted. The lengths of genetic elements are not drawn to scale. Since Foxp3 is exclusively expressed by Treg cells to drive cell fate and immune suppressive function, except for specific conditions like colonic Treg cells (35), examining these *Foxp3* enhancers offers a unique approach to explore the causal role of the induction efficiency of polyclonal Treg cells in immune tolerance. Pro, promoter. **(C)** Treg induction efficiency, which is linked to the probability of target coverage and autoimmune status, is assessed in mice deficient in *Aire*, CNS0, or CNS3, either individually or in combinations. The y-axis represents the relative severity (not drawn to scale) of autoimmune inflammation in specific-pathogen-free facilities.

Thymic precursor cells expressing TCRs with high reactivity to self-antigens or agonists undergo apoptosis (negative selection) and those with moderate reactivity differentiate into Treg cells in the presence of IL-2 by inducing the expression of Foxp3 and functional genes, such as CD25 and CTLA-4 (**Figure 2A**) (12, 44). CD4 naïve T cells can also differentiate into Treg cells in the periphery upon antigen stimulation under favorable conditions, such as IL-2, TGF-β, and retinoic acid (45–48). The TCR repertoire, once acquired during Treg induction, is inherited throughout Treg lifespan.

Treg induction, like other T-cell fate determination processes, exhibits considerable variations depending on the peptide antigens (13, 16, 17, 45, 49–52). Precursor cells expressing the same TCRs can be induced into Treg cells or remain as Tcon cells (**Figure 2A**). This aligns with the presence of overlapping TCRs in Treg and Tcon cells (15, 53, 54). Given that peptide antigens elicit a wide range of TCR signal strengths and peptide abundance varies significantly (31, 50, 52, 55, 56), Treg induction for individual peptide antigens would exhibit substantial variations. As a result, there would be considerable fluctuations on the coverage and representation of Treg cells across different peptide antigens. While experimental measurements are necessary to define this uncertainty, a critical question arises about how Treg cells can effectively control overrepresented Tcon cells to achieve robust tolerance.

## Immune tolerance and polyclonal Treg induction regulated by *Foxp3* enhancers

Studies of polyclonal Treg induction through the regulation of the *Foxp3* gene by its enhancers have provided a unique approach to assess the significance of varying Treg induction and target representation. This is because perturbations of these enhancers do not directly affect Treg function (**Figure 2B**) (13, 49, 57, 58). Two of these enhancers, designated as conserved non-coding sequences (CNS) CNS0 and CNS3, exert significant influence on the induction of both thymic and peripheral Treg cells. CNS0 senses the IL-2-STAT5 signaling, while CNS3 is involved in the NF-κB signaling (13, 49, 58, 59). They are also regulated by histone H3 lysine four methylation (13, 49, 59–61). CNS1 is a response element for the TGF-β-Smad signaling, mainly contributing to Treg induction in the periphery (49, 62).

Ablation of CNS0 or CNS3 alone in mice results in a moderate reduction of Treg induction (**Figure 2C**) (13, 49, 57, 58). This does not lead to Tcon activation or autoimmune responses, suggesting that the remaining Treg cells can effectively suppress Tcon cells. Deletion of both CNS0 and CNS3 further reduces Treg induction by approximately 100-fold, causing multiorgan autoimmune inflammation and a shortened lifespan comparable to *Foxp3* deletion (3, 4, 14, 59). Although the stability of the Treg lineage is also partially compromised, the significantly reduced target coverage and representation, ultimately determined by Treg induction, seem to play a major role (13, 14, 49, 59).

This is consistent with the additive effects of *Aire-*CNS0 *and Aire*-CNS3 double deficiencies. Nuclear protein Aire is expressed in medullary thymic epithelial cells (mTECs) to facilitate the expression of tissue-restricted self-antigens for thymic negative selection and Treg induction (63). Mice lacking *Aire* develop late-onset autoimmune disease (23). Combined deletion of *Aire* with CNS0 or with CNS3 drastically exacerbates the disease severity, along with a further reduction of Treg induction (**Figure 2C**) (13, 58).

These results collectively indicate that the efficient induction of polyclonal Treg cells is essential for effective suppression of autoreactive Tcon cells. Unsurprisingly, Treg cells expressing monoclonal TCRs or a reduced TCR repertoire exhibit limited suppressive capability (64, 65). However, the relative significance and capacity of antigen-specific and bystander suppressions of polyclonal Treg cells have not been defined by these studies.

## The suppression modes of Treg cells against specific antigens

The investigation of antigen-specific Treg cells has been hindered by their scarcity, identification, and manipulation in native conditions (16–18). In addition, Treg cells are closely connected to other tolerance programs, such as thymic negative selection, anergy, and exhaustion of Tcon cells, making it difficult to interpret the results when Treg cells are perturbed. For instance, Shin et al. demonstrated that self-antigens exposed during lung injury induce Treg cell expansion, but the ablation of Treg cells does not result in Tcon cell proliferation. This suggests that Tcon cell anergy or exhaustion occurs in this context (66).

**Figure 3:**
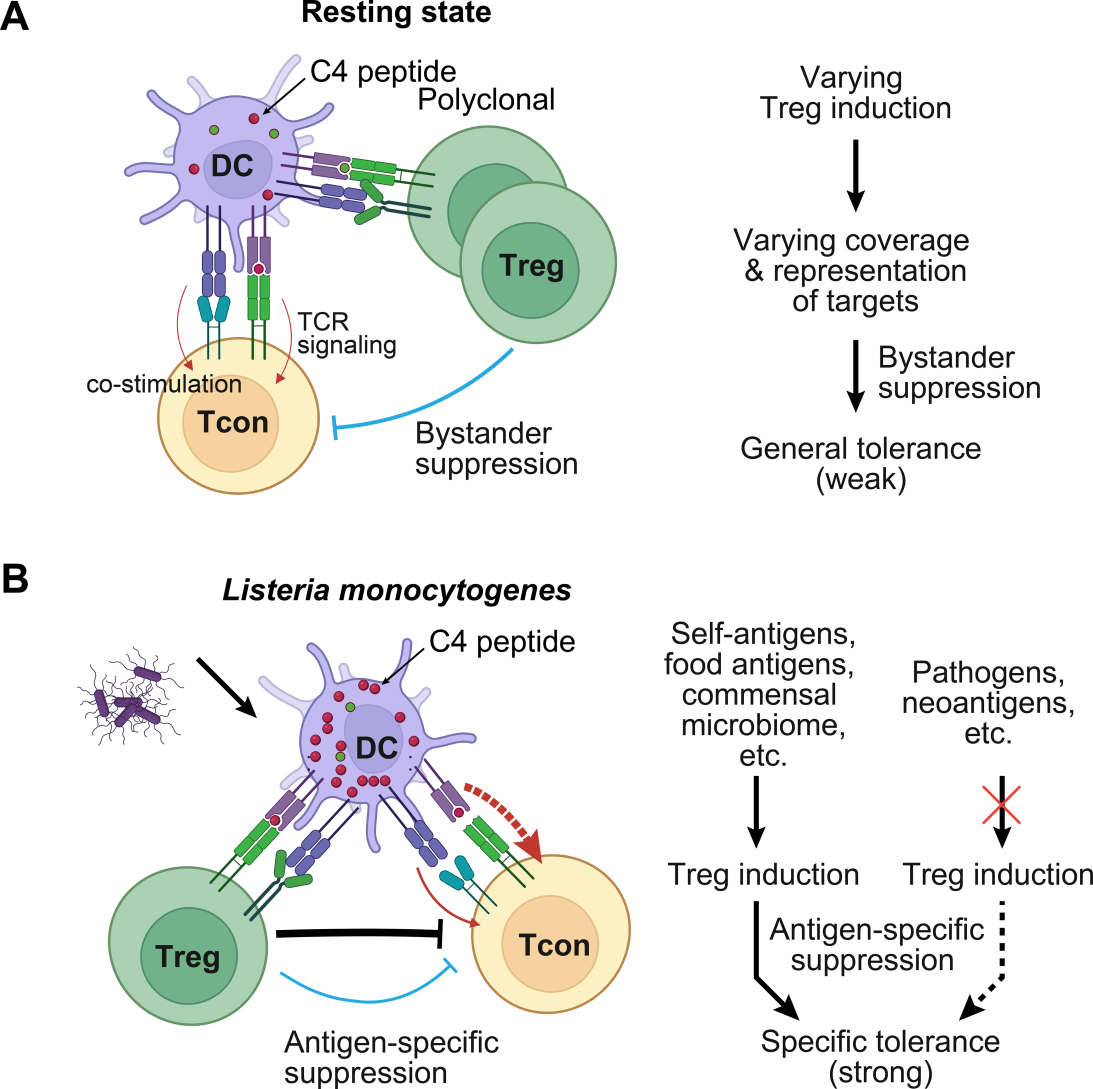
Evaluation of the suppression modes of antigen-specific Treg cells. **(A)** Klawon et al. recently employed MJ23 TCR transgenic mice and conditional deletion of the C4 epitope of prostate antigen TCAF3 in mTECs to demonstrate that thymic Treg cells against the C4 peptide are dispensable for suppressing related TCR-transgenic Tcon cells in the otherwise wild-type background (20). However, when polyclonal Treg cells are depleted, mice develop prostatitis, which is mediated by Tcon cells. These findings indicate that polyclonal Treg cells can effectively suppress C4-specific MJ23 Tcon cells within the same microenvironment through bystander suppression. A schematic model illustrates how general T-cell immune tolerance is maintained in the resting state to oppose inherent variations in Treg induction and target coverage or representation. **(B)** When the C4 peptide is delivered to mice via *Listeria monocytogenes*, presumably at a higher level, C4-specific Treg cells are now indispensable for suppressing MJ23 Tcon cells (20). Mice lacking C4-specific Treg cells fail to restrain MJ23 Tcon cells after infection with *L. monocytogenes* expressing C4 peptide, despite the presence of bystander suppression from polyclonal Treg cells. Consequently, Treg antigen-specific suppression plays a dominant role in conferring tolerance to particular targets that induce Treg cells during previous exposure. This mechanism ensures that the inflammatory response of Tcon cells to other abundant antigens, such as pathogens and neoantigens, remains unaffected in the absence of matched Treg cells.

Klawon et al. examined prostate-specific Treg and Tcon cells against the C4 peptide of transient receptor potential channel–associated factor 3 (TCAF3) in mice (20). C4 is presented by mTECs via MHC II to induce Treg cells without causing significant negative selection (67). Whereas ablation of all Treg cells results in prostatitis, depletion of C4-specific Treg cells through conditional deletion of C4 in mTECs does not activate Tcon cells expressing C4-specific MJ23 transgenic TCR, either in the steady state or after innate immune activation by LPS, poly(I:C), or *L. monocytogenes* infection. Thus, polyclonal Treg cells can successfully suppress Tcon cells with an unrelated antigen specificity in this context (**Figure 3A**).

When C4 peptide is delivered via *L. monocytogenes*, presumably at a higher level, C4-specific Tcon cells can be fully suppressed in wild-type mice but not in mice lacking C4-specific Treg cells (20), indicating a crucial role of antigen-specific suppression by Treg cells (**Figure 3B**). Likewise, Tcon cell response to LLO_190–201_ epitope derived from *L. monocytogenes* is not affected by C4-specific Treg cells (20).

Antigen load appears to be a key determinant of the relative significance of antigen-specific and bystander suppression modes of Treg cells (20, 68). At low levels of antigens, bystander suppression by polyclonal Treg cells is sufficient to control unrelated Tcon cells. However, when the antigen level is high, antigen-specific suppression becomes dominant.

## Treg suppression modes related to antigen load and resulting TCR signal strength

In contrast to Klawon et al.’s observations, the deletion of insulin expression in mTEC in mice impairs the generation of insulin-specific Tregs, resulting in autoimmune diabetes (69). These differences could be attributed to additional factors, such as varying antigen levels, TCR signal strengths, persistence of antigen stimulation, or the nature of tissue environments including APCs. Besides, self-antigens presented at higher levels postnatally often disrupt established immune tolerance, presumably by surpassing antigen-specific Treg suppression (70, 71). For instance, tyrosinase-related proteins (TRPs) TRP-1 and TRP-2 when expressed via vaccinia virus induce an autoimmune attack on melanocytes in mice (72). Immunization of animals with high levels of self-antigens expressed by the central nervous system induces multiple sclerosis (73, 74).

**Figure 4:**
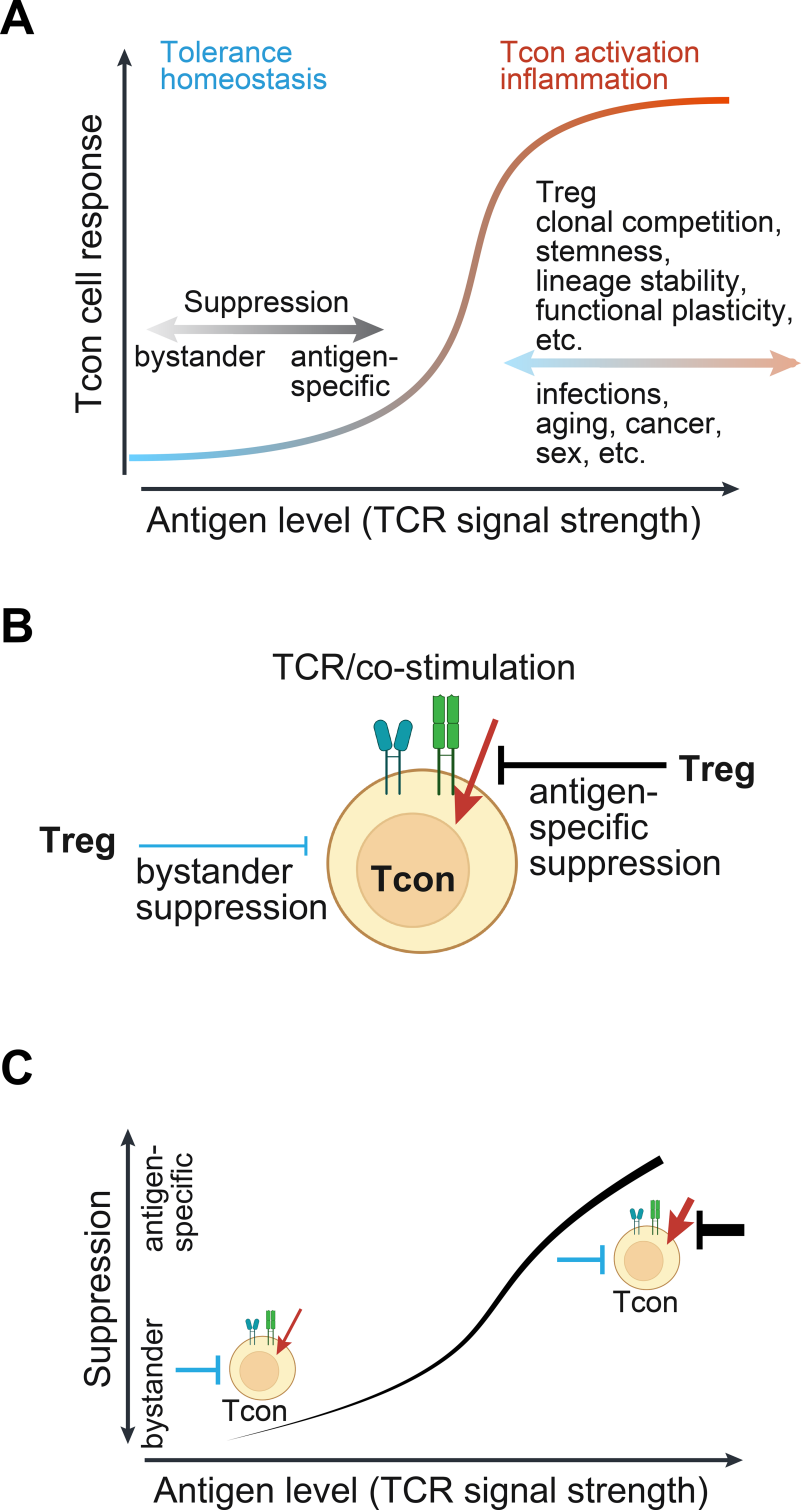
The relativity of Treg suppression modes and tolerance capacity. **(A)** An integrated model illustrates the causal role of relative antigen load and the resulting TCR signal strength. Antigen load is defined as the relative levels of specific peptide antigen or epitope that binds to corresponding TCRs to induce signal transduction above the tonic or basal level. The TCR signal strength is reflected by the magnitude of downstream signal transduction, gene expression, and T-cell activity depending on cell types. The antigen load or TCR signal strength determines the relative importance of suppression modes in maintaining immune tolerance, which is exhibited following Treg deficiency. As antigen level increases, Treg antigen-specific suppression is gradually surpassed, thereby unleashing Tcon cells to initiate inflammatory responses. This response curve may be modified by Treg and Tcon intrinsic and extrinsic factors. Several such factors are listed. **(B)** The positive and negative signals acting on Tcon cells. For simplicity, these signals are not depicted in detail. The positive signals (red) from TCR and co-stimulatory receptors are countered by Treg antigen-specific (black) and bystander (blue) suppression mechanisms, which may be separated in space and time. The relative weights of negative and positive signals determine the outcome of Tcon cells, namely non-reactivity or inflammatory response. **(C)** The relative significance of Treg suppression modes when antigens are presented at varying levels. This model applies to Treg cells that recognize a specific antigen in the presence of polyclonal Treg cells. Line thickness represents the signal strength.

Although the precise causes of these different outcomes remain to be determined, a general relationship emerges between antigen load, which determines the strengths of TCR signaling, and the suppression mechanisms, capacity, and extent of immune tolerance demonstrated by Treg cells (**Figure 4A**). The capacity of antigen-specific suppression of Treg cells is established during Treg induction, reflected by the target representation. When antigen levels are relatively low, bystander and antigen-specific suppressions can effectively maintain immune tolerance. However, as antigen load increases, there is a greater reliance on antigen-specific suppressions. However, excessive antigen stimulation can overwhelm Treg suppressive capacity. If the corresponding Tcon cells are not in non-functional states, such as anergy and exhaustion (66, 75, 76), they would become activated and trigger inflammatory responses.

This response curve may be shifted by additional factors that modulate Treg and Tcon cells, such as antigen persistence that impacts Treg and Tcon cells differentially and innate signals that favor Tcon cells and inflammatory response.

## Treg suppression and antigen stimulation are weighted by Tcon cells

By analyzing the input signals and outcomes from the perspective of Tcon cells, we can gain a deeper understanding of Treg suppression modes. Treg suppression and antigen stimulation are negative and positive regulators, respectively (**Figure 4B**). They may act on Tcon cells separately in space and time. Their relative weights determine whether Tcon cells are ultimately activated to initiate an inflammatory response. At low levels of antigen stimulation, weak TCR signaling in Tcon cells can be readily overridden by Treg bystander suppression (**Figure 4C**). However, the potency of anti-inflammatory molecules secreted by Treg cells is limited. When antigens are abundant and TCR signaling is potentially high on Tcon cells, Treg cells predominantly suppress Tcon cells in an antigen-specific manner, primarily by restricting antigen stimulation. High-affinity TCRs, a pre-active state, and depletion of co-stimulatory receptors CD80/CD86 and peptide-MHC II complexes on APCs enable Treg cells to outcompete Tcon cells during antigen stimulation (12, 31, 36, 38, 39). Notably, depleting IL-2 (IL-2 sink) or secretion of TGF-β by Treg cells as bystander suppression mechanisms can downregulate TCR signaling in Tcon cells (77–80). A further increase in antigen load and TCR signaling would eventually overpower Treg suppression, leading to the activation of Tcon cells, unless other tolerance programs are induced in the meantime (**Figure 4A**). Innate signals and proinflammatory cytokines, although present in low concentrations initially, may promote Tcon polarization and the inflammatory response.

How TCR signaling and Treg suppression are mechanistically integrated by Tcon cells remains to be fully elucidated. *In vivo* visualization of the proximity of Treg, Tcon, and APCs has yielded valuable insights (20, 81). Treg bystander suppression does not impact Tcon cell priming, indicating that it operates at a later stage, such as during stimulation-induced Tcon cell proliferation (20). In contrast, Treg antigen-specific suppression seems to exert its effects through both Tcon cell priming and proliferation. The distinct outcomes reflect the quantitative differences in TCR signal strengths involved in different suppression mechanisms. This process may also be influenced by various factors related to infection, aging, cancer, and sex through antigen presentation and the function of Treg and Tcon cells.

## Discussion

Extensive research conducted over the past two decades has revealed increasing complexities on Treg suppression modes, applicable conditions, and constraints. Here, we propose a unified model that illustrates how the relative significance and capabilities of Treg bystander and antigen-specific suppression modes are shifted by the levels of cognate antigens and resulting TCR signal strengths. It distills the intricate interactions among Treg target coverage and representation, Tcon reactivity, APCs, antigen load, and TCR signal strength. When applicable, additional modulators can be readily incorporated into this model.

Our simplified formula facilitates further investigation of the fundamental principles and mechanisms underlying Treg-mediated immune tolerance, such as how robust T-cell tolerance is established and maintained by Treg cells that have inherent variations in target coverage and representation, how immune tolerance is disrupted when Tcon cells surpass Treg suppression to cause autoimmune inflammation upon genetic and environmental perturbations, and how immune homeostasis and tolerance can be reversed by Treg cells following autoimmune dysregulation. Our model also helps elucidate the modulators of Treg suppression mechanisms, including the incorporation of new Tregs, clonal competition, lineage stability, and functional plasticity. These variables remain poorly defined due to technical challenges.

Treg cells hold great promise for therapeutic applications (82–85). Exploration of our formula may lead to new strategies on reducing Treg suppression for cancer treatment while maintaining tolerance to normal tissues, if the antigen levels vary and the relative significance of Treg suppression modes differ in these two settings. It may facilitate the development of innovative methodologies for utilizing polyclonal or antigen-specific Treg cells to treat immunological diseases. The strategies can be tailored based on the levels of relevant antigens and the desired potency of suppression. For instance, experimental autoimmune encephalomyelitis can be mitigated by both antigen-specific and bystander suppression modes of Treg cells introduced via noninflammatory mRNA vaccine (71). Furthermore, Treg function can be selectively enhanced beyond the natural capacity through various approaches, such as directing Treg cells to specific antigens via TCRs or chimeric antigen receptors, enhancing Treg lineage stability and fitness, or increasing suppressive function. Our model also offers a simple framework for designing and evaluating engineered Treg cells.

## Model limitations and other hypotheses

This theoretical analysis assumes that the Treg representation established during Treg induction is not compensated by clonal expansion based on antigen levels at the Treg functional stage. However, a certain degree of proliferation could be involved, allowing for a range of antigen-level increases. Experimental measurements are necessary to test these possibilities and identify the factors limiting Treg clonal expansion and functionality.

Our current model simplifies the analysis by treating thymic-derived Treg cells as a homogeneous population. This model would become more complex in various contexts involving different antigen levels, response curves, mechanisms and magnitudes of Treg bystander and antigen-specific suppressions, as well as the numbers, reactivity, and effector functions of target Tcon cells. Treg cells also regulate many other cell types, a topic not covered in this paper.

This model may also apply to peripheral Treg cells, but exhibit distinct response curves due to varying antigen load and regulatory mechanisms (2). For instance, IL-10 plays a more significant role in Treg cells suppressing inflammation at the mucosal interface (8, 86). Antigens in food and the commensal microbiota are often presented at high levels (87, 88), leading to a primary reliance on antigen-specific suppression by Treg cells. A high representation of these Treg cells would be crucial for adequate tolerance.

Treg cells differentiate into various subsets, including tissue-resident Treg cells, Th1-like Tregs, Th2-like Tregs, follicular Treg (Tfr) cells, and Th17-like Treg cells (1, 2, 89). These Treg cells suppress Tcon differentiation and function under special environments through additional mechanisms. The same principles discussed in this paper would govern their suppression modes. The relative importance of antigen-specific and bystander suppressions would vary due to quantitative differences in suppressive molecules, such as the levels of CD25, IL-10, and CTLA-4, as well as the activity of Tcon cells.

Furthermore, the tissue repair function of Treg cells, such as the secretion of amphiregulin (Areg) upon IL-18 or IL-33 stimulation (90), may be comparable to Treg bystander suppression. However, the connection with antigen-specific Treg function remains to be fully elucidated.

Further experiments conducted under these conditions will test the model’s applicability and quantify the variables and modifiers involved. This would provide deeper mechanistic insights and strengthen the principles underlying complex Treg function.

## Data Availability

The original contributions presented in the study are included in the article/supplementary material. Further inquiries can be directed to the corresponding author.
